# Secular Trends of Liver Cancer Mortality and Years of Life Lost in Wuhan, China 2010–2019

**DOI:** 10.3390/curroncol30010071

**Published:** 2023-01-09

**Authors:** Yuanyuan Zhao, Donghui Yang, Yaqiong Yan, Xiaoxia Zhang, Niannian Yang, Yan Guo, Chuanhua Yu

**Affiliations:** 1 Wuhan Center for Disease Control and Prevention, Wuhan 430024, China; 2 Department of Epidemiology and Biostatistics, School of Public Health, Wuhan University, Wuhan 430072, China

**Keywords:** liver cancer, disease burden, trend, decomposition, Wuhan

## Abstract

Background: Liver cancer has caused a heavy burden worldwide. This study aimed to estimate the trends in the mortality and years of life lost (YLL) due to liver cancer and decompose the total deaths into three contributors: population growth, population aging, and mortality change. Methods: Our study used data from the cause-of-death surveillance system in Wuhan. The mortality and YLL rates were standardized according to the sixth national population census in China. This study calculated the estimated annual percentage change (EAPC) to estimate the trends in the age-standardized mortality rate (ASMR) and age-standardized YLL rate (ASYR). Meanwhile, a decomposition analysis was used to explore the effect of population growth, population aging, and age-specific mortality change on the change in liver cancer deaths. Results: The ASMR of liver cancer declined at an annual rate of 4.6% from 30.87 per 100,000 people in 2010 to 20.29 per 100,000 people in 2019, while the ASYR was at an annual rate of 5.6% from 969.35 per 100,000 people in 2010 to 581.82 per 100,000 people in 2019. Similar downward trends were seen in men and women. The decomposition analysis found that total deaths number changed by −12.42% from 2010 to 2019, of which population growth and population aging caused the total death numbers to increase by 9.75% and 21.15%, while the age-specific mortality change caused the total death numbers to decrease by 43.32%. Conclusion: Although the ASMR of liver cancer has declined in recent years in Wuhan, it still causes a heavy burden with the increasing population and rapid population aging and remains an essential public health issue. The government should take measures to reduce the burden of liver cancer, especially among men.

## 1. Introduction

Liver cancer causes a heavy burden globally. It was estimated that approximately 830,000 liver cancer deaths occurred in 2020, which accounted for 8.3% of all cancer deaths [1]. Liver cancer is the third leading cause of cancer-related death worldwide, following lung cancer (18.0%) and colorectal cancer (9.4%). Previous studies found that the hepatitis B virus (HBV) was the main risk factor for liver cancer in developing countries, while the hepatitis C virus (HCV) and alcoholic drinking were the essential risk factors for liver cancer in developed countries [2,3,4]. The epidemic characteristics of liver cancer vary significantly across several global regions. Compared with other regions, liver cancer is more common in Asia, especially in China [5]. In 2015, approximately 326,000 liver cancer patients died in China, and the mortality rate was calculated as 15.33 per 100,000 people [6]. In 2020, there were 391,000 estimated liver cancer deaths in China, which accounted for half of global liver cancer deaths. Furthermore, it was predicted that liver cancer death would increase to 572,000 in 2040 in China [1]. The global burden of liver cancer, especially in China, currently remains serious.

Meanwhile, previous studies have demonstrated that the burden of liver cancer is largely determined by the years of life lost (YLL). The YLL accounts for the death count and life expectancy at death, which could help set priorities for prevention and compare the premature mortality experience between populations. Estimating the YLL plays an essential role in the study of the global burden of disease. In 2019, liver cancer caused 12.5 million DALY, of which the YLL accounted for 99.0%, and the YLD accounted for 1.0% [7]. 

Many factors could contribute to the increase in the liver cancer burden. Briefly, the change in liver cancer deaths could be attributed to population growth, population aging, and age-specific mortality change. The population worldwide has increased rapidly in the last decades, and it was estimated that the population would reach approximately 8 billion in 2022, 8.5 billion in 2035, 9.7 billion in 2050, and 10.4 billion in 2100 [8]. Meanwhile, population aging is also developing rapidly as a result of socioeconomic development. The number of people aged 60 and above was 962 million in 2017, and it is predicted to be 2.1 billion in 2050 and 3.1 billion in 2100 [8]. These factors may increase the number of liver cancer deaths. However, the prevention and treatment of liver cancer may improve age-specific mortality and decrease the burden of liver cancer. Their separate effects on the change in liver cancer deaths are unclear.

Wuhan is one of the largest cities in China, with an area of 8594 square kilometers and a population of over 12.32 million. In the past years, the government has tried to reduce the burden of liver cancer. However, the trend in the burden of liver cancer remains unknown. Therefore, this study aimed to explore the age-standardized mortality rate (ASMR) trend and the age-standardized YLL rate (ASYR) trend of liver cancer in Wuhan. Furthermore, this study also decomposed the change in liver cancer and investigated the separated effect of population growth, population aging, and age-specific mortality change.

## 2. Materials and Methods

### 2.1. Study Population

The mortality data in this study were obtained from the cause-of-death surveillance system at the Wuhan Center for Disease Control and Prevention. The information included age, sex, cause of death, and date of death, which was further adjusted through garbage code cleaning. Liver cancer was defined with the ICD-10 code from the 2019 GBD (C22–C22.4, C22.7–C22.9, and Z85.05). Finally, we extracted the liver mortality data among 13 age groups of 5-year intervals (20–24, 25–29, 30–34, 35–39, 40–44, 45–49, 50–54, 55–59, 60–64, 65–69, 70–74, 75–79, and 80–84 years) from 2010 to 2019. Informed consent forms were not required as the death data information originated from the monitoring system. The study was approved by the ethics committee of the Wuhan Center for Disease Control and Prevention. The population data in Wuhan was extracted from the Wuhan public security system. Meanwhile, this study used the population census in 2010 in China as a standardized population to estimate the age-standardized rate.

### 2.2. Statistical Analysis

First, this study calculated the liver cancer mortality rates among both sexes and subgroups stratified by sex between 2010 and 2019. In addition, this study estimated the YLL of liver cancer, which was a metric of premature death calculated as the sum of the death count multiplied by the standard life expectancy at each age. The standard life expectancy was obtained from the 2017 GBD (https://ghdx.healthdata.org/record/global-burden-disease-study-2017-gbd-2017-reference-life-table, accessed on 24 February 2022). To eliminate the effect of population structure, the ASMR and ASYR were computed by using the direct population normalization method of the sixth national population census.

Generally, the age-standardized rate (ASR) trends reflect the change in disease patterns and the effectiveness of the current prevention strategies within a population. Currently, the estimated average percentage change (EAPC) and corresponding 95% confidence interval (CI) are widely used indicators to evaluate ASR trends in epidemiological studies [9]. The EAPCs and their 95% confidence intervals (CIs) were obtained from Joinpoint (version 4.8.0.1). The ASR showed a significant upward trend if the EAPC and it’s lower bound of the 95% CI were both >0. The ASR showed a significant downward trend if both the EAPC and it’s upper bound of the 95% CI were both <0. Otherwise, the trend of the ASR was considered to be stable.

Meanwhile, based on methods developed by Das Gupta [10], we conducted a decomposition analysis and divided the change in liver cancer deaths from 2010 to 2019 into three explanatory components: total population growth, population aging, and the age-specific mortality rate of liver cancer [11]. We calculated a separate effect on the change in liver cancer deaths from 2010 to 2019.

SAS (version 9.4) and Python (version 3.7) were used for the data processing. The EAPC was estimated in Joinpoint (version 4.8.0.1). The decomposition analysis was conducted in R (version 4.0.2). Statistical significance was considered when two-sided *p* < 0.05.

## 3. Results

**Figure 1:**
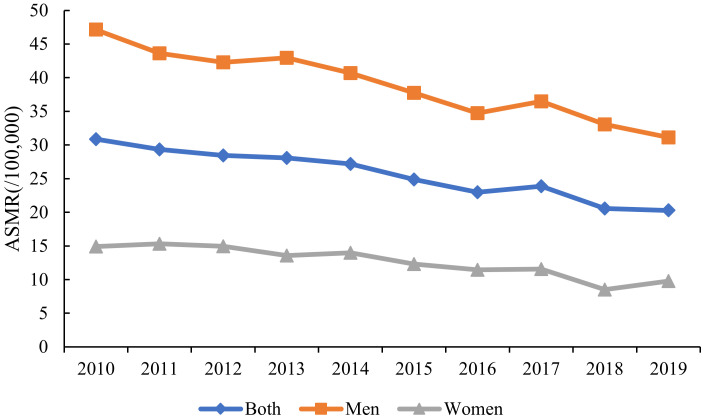
The Trends in the ASMRs of Liver Cancer in Wuhan from 2010 to 2019.

Figure 1 and Table S1 shows the trend in the ASMR of liver cancer in Wuhan from 2010 to 2019. During the period of 2010–2019, the overall trend showed an obvious decline. In 2010, the ASMR in Wuhan was 30.87 per 100,000 people. In 2019, it had dropped to 20.29 per 100,000 people, a relative decrease of 34.38%. Moreover, the ASMR of liver cancer in men from 2010 to 2019 was generally higher than those in women, and the ASMR of liver cancer in men and women also showed a downward trend during these years. The ASMR of liver cancer decreased from 47.15 per 100,000 people in 2010 to 31.13 per 100,000 people in 2019 by 33.98% in men, while the ASMR of liver cancer decreased from 14.9 per 100,000 people in 2010 to 9.78 per 100,000 people in 2019 by 34.37% in women.

**Figure 2:**
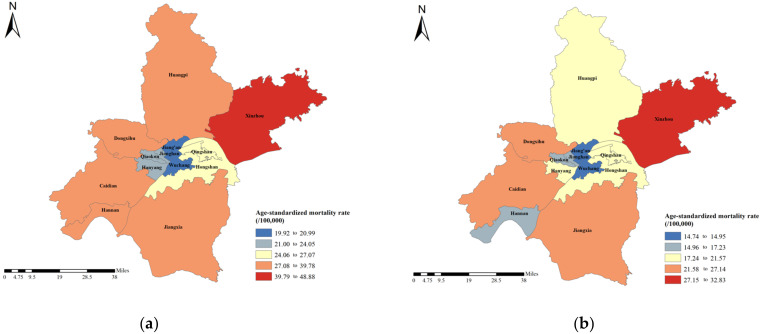
The ASMRs of liver cancer in Wuhan in (**a**) 2010 and (**b**) 2019.

Figure 2 and Table S2 shows the ASMRs of districts in Wuhan in 2010 and 2019. Xinzhou, Hannan, and Caidian showed the highest ASMR in 2010, with an ASMR of 48.88 per 100,000 people, 39.78 per 100,000 people, and 39.41 per 100,000 people, respectively. In 2019, the top three highest districts were Xinzhou, Caidian, and Jiangxia, with an ASMR of 32.83 per 100,000 people, 27.14 per 100,000 people, and 25.54 per 100,000 people, respectively.

**Figure 3:**
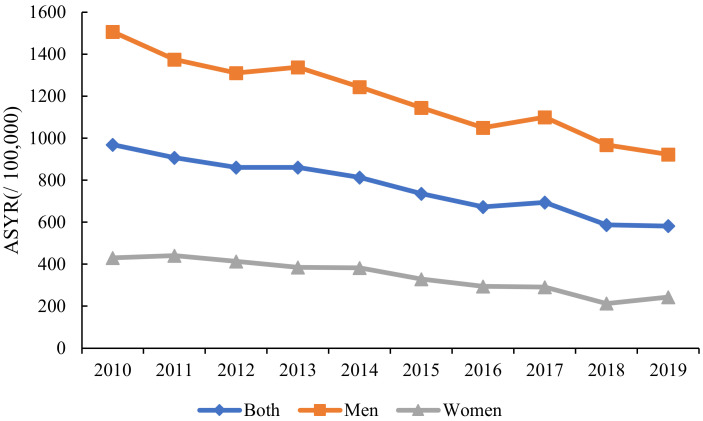
The Trends in the ASYRs of Liver Cancer in Wuhan from 2010 to 2019.

As for the ASYR of liver cancer, Wuhan showed an overall trend of steady decline from 2010 to 2019 in Figure 3. In Wuhan, the ASYR of liver cancer was 969.35 per 100,000 people in 2010, and it declined to 581.82 per 100,000 people in 2019, a relative decrease of 39.98%. Similar to the trend in the ASMR, the ASYR of liver cancer also decreased among men and women. From 2010 to 2019, the ASYR in men decreased from 1507.56 per 100,000 people to 923.01 per 100,000 people, a relative decrease of 38.77%. The ASYR decreased from 430.50 per 100,000 people in 2010 to 243.48 per 100,000 people in 2019 in women, a relative decrease of 43.44% from 2010 to 2019. Meanwhile, it was observed that the ASYR of liver cancer in men was higher than that of women from 2010 to 2019, indicating that the burden of liver cancer in men was severe.

**Figure 4:**
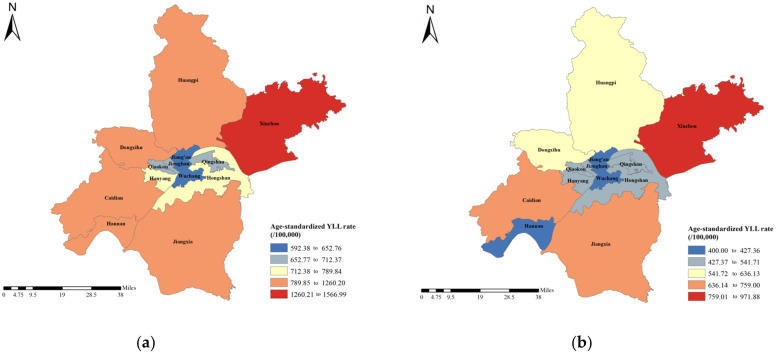
The ASYRs of liver cancer in Wuhan in (**a**) 2010 and (**b**) 2019.

Figure 4 shows the ASYRs of districts in Wuhan in 2010 and 2019. Xinzhou, Caidian, and Hannan showed the highest ASYRs in 2010, with an ASYR of 1566.99 per 100,000 people, 1260.20 per 100,000 people, and 1251.97 per 100,000 people, respectively. In 2019, the top three highest districts were Xinzhou, Jiangxia, and Caidian, with ASYRs of 971.88 per 100,000 people, 759.00 per 100,000 people, and 732.60 per 100,000 people, respectively.

**Figure 5:**
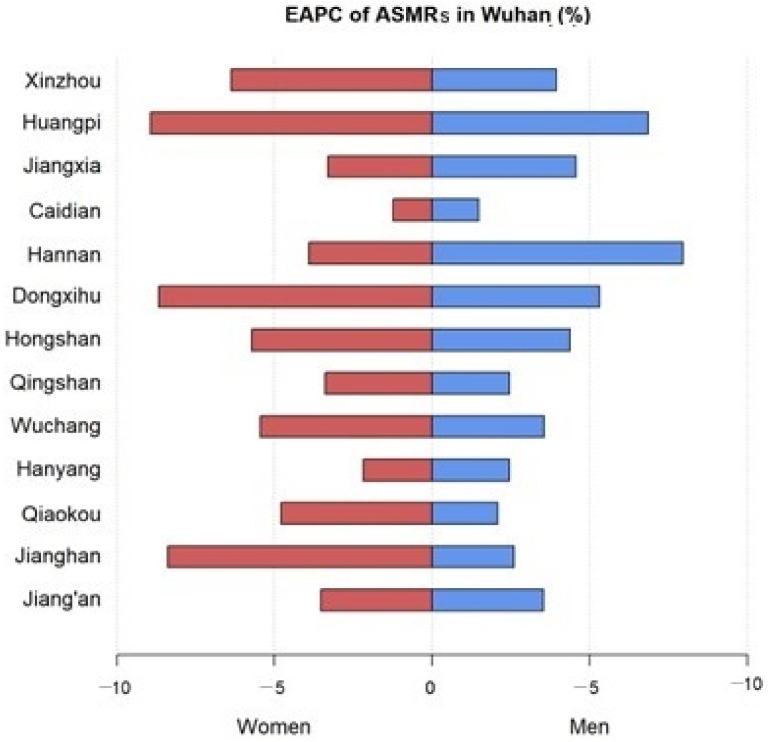
EPACs of ASMRs among the women and men in the districts of Wuhan.

The EAPCs of the ASMRs from 2010 to 2019 in Wuhan are shown in Table 1 and Figure 5. The ASMR of liver cancer in Wuhan decreased significantly at an annual rate of 4.23% in men (EAPC = −4.23, 95% CI: −4.90 to −3.55), 5.77% in women (EAPC = −5.77, 95% CI: −7.40 to −4.12) and 4.6% in both sexes (EAPC = −4.64, 95% CI: −5.36 to −3.91) between 2010 and 2019. Huangpi, Hannan, and Xinzhou showed a rapid decline from 2010 to 2019, with EAPCs of −7.31 (95% CI: −9.61 to −4.95), −7.25 (95% CI: −10.00 to −4.41), and −6.11 (95% CI: −7.30 to −4.91), respectively. Most of the districts showed a faster decrease in the ASMRs in women.

**Figure 6:**
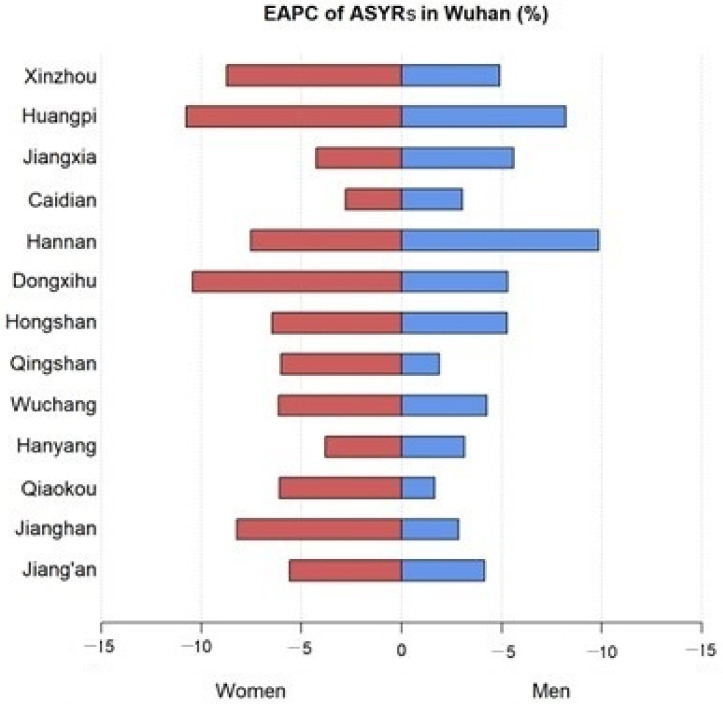
EPACs of ASYRs among women and men in the districts of Wuhan.

Similar to the ASMR, the ASYR also showed a significant downward trend in Wuhan (Table 1 and Figure 6). The ASYR of liver cancer in Wuhan decreased significantly in men (EAPC =−5.05, 95% CI: −5.78 to −4.33), women (EAPC = −7.54, 95% CI: −9.20 to −5.86), and both sexes (EAPC = −5.63, 95% CI: −6.39 to −4.85) during 2010 to 2019. Hannan, Xinzhou, and Dongxihu declined at an annual rate of 9.68% (EAPC = −9.68, 95% CI: −12.36 to −6.91), 8.76% (EAPC = −8.76, 95% CI: −11.08 to −6.38), and 6.41% (EAPC = −6.41, 95% CI: −7.37 to −5.45), respectively. Most of the districts showed a faster decrease in the ASYR in women.

Table 2 shows the results of the decomposition analysis, which includes the effects of population growth, population aging, and the age-specific mortality rate change. From 2010 to 2019, the total number of deaths caused by liver cancer in Wuhan changed by −12.42%. The effect of population growth, population aging, and age-specific mortality change caused a 9.75%, 21.15%, and −43.32% change in the total death number, respectively. Among men, the total number of deaths caused by liver cancer in Wuhan changed by −13.86%, while the effects of population growth, population aging, and the change in age-specific mortality caused a 9.13%, 19.40%, and −42.39% change, respectively. Among women, the total number of deaths caused by liver cancer in Wuhan changed by −8.06%, while population growth, population aging, and the change in age-specific mortality caused a 10.39%, 25.55%, and −44.00% change, respectively.

## 4. Discussion

From 2010 to 2019, the ASMRs and ASYRs of liver cancer in Wuhan decreased significantly among men, women, and both sexes. The disease burden of liver cancer in men was higher than that in women from 2010 to 2019. This study found that population growth and aging led to an increase in liver cancer deaths, while the improvement of the age-specific mortality of liver cancer contributed to a decline in the death numbers.

A heavier disease burden of liver cancer in men was also found in other studies. In 2015, the number of liver cancer deaths in men was nearly three times that in women in China [6]. There were discrepancies in many risk factors, which contributed to the discrepancy between men and women. Viral infection was the main cause of liver cancer. It was estimated that the prevalence of HBV infection in men was 5.88% and higher than in women (5.05%) [12]. This may have partly resulted in the discrepancy in liver cancer burdens between men and women. Besides viral infections, risk factors, such as alcohol drinking and smoking, could also contribute to the increase in liver cancer [13]. In 2012, the prevalence of alcoholic drinking was 13.9% in women [14] and 57.8% in men [15]. In addition, previous studies showed that the prevalences of previous and current smokers in men were 67.39 and 48.77%, which were higher than those in women (3.74 and 2.93%) [16]. Besides viral infection and traditional modifiable risk factors that could be prevented, sex hormones could also have a crucial role in the pathogenesis and development of HBV-induced liver cancer [17]. Therefore, the disease burden of liver cancer was higher in men than in women. More attention should be paid to men who have high risks of liver cancer.

The disease burden of liver cancer declined steadily from 2010 to 2019 in men and women, which was partly due to vaccination, reducing the incidence of liver cancer. A previous study conducted in Taiwan showed that the incidence of liver cancer was four times higher in people who did not receive vaccination than those who received it [18]. In the past years, vaccination coverage has increased gradually in China. It was reported that the coverage of three doses of the hepatitis B vaccine for infants was 30.0% in 1992 and increased to 99.6% in 2015, and timely birth-dose coverage increased from 22.2% in 1992 to 95.6% in 2015 [19,20]. Furthermore, China not only paid much attention to infant vaccination, but from 2009 to 2011, China implemented a vaccination program to strengthen the protection against hepatitis B infection, and approximately 68 million adolescents under the age of 15 were vaccinated [21]. These measures could decrease the incidence of hepatitis B disease and further reduce the burden of liver cancer. GLOBOCAN predicted the increasing death numbers of liver cancer in China, but liver cancer in Wuhan showed a declining trend. The difference may be that liver cancer in Wuhan was controlled better than the average level in China, which led to the decreased death number in Wuhan in contrast to the increasing death numbers in China in the near future. Compared with men, the ASMR and ASYR declined faster in women, partly because the government has paid much attention to the prevention of the mother-to-child transmission of HBV [22].

Meanwhile, previous studies showed that liver cancer was mostly in the advanced examination stage and could only live for a few months [23]. Advanced early detection technology is essential to reduce the burden of liver cancer. With the development of society, detection technology also updates gradually. In addition to traditional screening methods, such as ultrasound imaging, computer tomography imaging, magnetic resonance imaging, and the measurement of serum alpha-fetoprotein levels [24], many recent methods, such as improved imaging techniques [25] and other candidate biomarkers, could help screen for liver cancer [26,27,28]. China’s central government implemented “early detection and early treatment of cancer in rural cancer” in 2005 [29] and the “cancer screening program in urban China” in 2012 [30]. These measures may partly decrease the burden of liver cancer.

Traditionally, medications, such as sorafenib, were used to treat liver cancer patients [31]. In recent years, researchers have started to concentrate on new molecularly targeted therapies, immunotherapies, and metabolism therapies. These would also prolong the life of liver cancer patients. From 2003 to 2015, it was estimated that the 5-year survival rate increased from 10.1% to 12.1% [32]. Although the situation of liver cancer in China improved, it was still severe compared with western countries. From 2006 to 2012, the 5-year survival rate of liver cancer was 21.0% in the US [33].

Our study found that remote districts, such as Xinzhou and Caidian, had high ASMRs and ASYRs. A previous study found that the ASMR of liver cancer was higher in lower gross domestic product per capita areas [34]. In China, there were regional disparities in medical resources [35]. Better medical resources were in more developed areas. Although districts such as Xinzhou and Caidian showed a faster decline, the burden of liver cancer was still heavy in these remote areas. Therefore, these areas should strengthen the measures to reduce the burden of liver cancer.

In the results of mortality decomposition, the age-specific mortality rate contributed to the decline of liver cancer deaths, while population growth and population aging led to an increase in liver cancer deaths. It reflected the effective results of the government’s measures to reduce the burden of liver cancer. Although age-specific mortality improved and further reduced the burden of liver cancer, population growth and aging may increase the burden of liver cancer. A previous study showed that the percentage of the population ≥ 65 years old increased in the last decades and continues until 2050 [36]. Compared with young adults, older people would have a high risk for liver cancer mortality [37]. With population growth, liver cancer incidence is still at a high level. Nearly half of all deaths caused by liver cancer occurred in China [1]. The WHO proposed the goal for the elimination of hepatitis B as a public health threat by 2030 [38]. China has continued to try to decrease the burden of liver cancer. For example, China has updated the guidelines for the stratified screening and surveillance of primary liver cancers [39]. Meanwhile, experts in China have achieved a consensus on the early screening strategies for liver cancer [40]. 

This study estimated the mortality and YLL rate trends of liver cancer and investigated the separate effects of population growth, population aging, and age-specific mortality, there were still some limitations in our study. First, we excluded the population aged 0–20 years and 85 years or older due to few deaths in Wuhan. Second, the results in this study are limited to Wuhan and, therefore, may not be applicable to other provinces.

## 5. Conclusions

In conclusion, although a significant downward trend in the ASMRs and ASYRs of liver cancer was observed in Wuhan due to population growth and aging, it remains an essential public health problem. More efforts should be performed to develop tailored prevention and control strategies aiming to reduce premature deaths from liver cancer in high-risk populations.

## Figures and Tables

**Table 1:** The ASMRs, ASYRs, and EAPCs of liver cancer from 2010 to 2019.

Characteristics	ASMR per 100,000 No. (95% UI)		EAPC	ASYR per 100,000 No. (95% UI)		EAPC
	2010	2019	No. (95% CI)	2010	2019	No. (95% CI)
Overall	32.23	20.29	−4.64	969.35	581.82	−5.63
	(29.56, 32.23)	(19.34, 21.3)	(−5.36, −3.91)	(961.84, 976.9)	(576.45, 587.25)	(−6.39, −4.85)
Sex						
Men	47.15	31.13	−4.23	1507.56	923.01	−5.05
	(44.84, 49.55)	(29.44, 32.93)	(−4.90, −3.55)	(1494.29, 1520.93)	(913.40, 932.75)	(−5.78, −4.33)
Women	14.9	9.78	−5.77	430.5	243.48	−7.54
	(13.64, 16.26)	(8.89, 10.80)	(−7.40, −4.12)	(423.50, 437.58)	(238.79, 248.31)	(−9.20, −5.86)
Regions						
Jiang’an	20.99	14.95	−3.55	652.76	427.36	−4.49
	(17.68, 24.88)	(12.42, 18.74)	(−6.12, −0.92)	(633.21, 672.87)	(412.57, 443.16)	(−7.00, −1.91)
Jianghan	22.65	16.51	−3.61	695.70	494.09	−3.70
	(18.42, 27.74)	(13.27, 21.64)	(−6.79, −0.31)	(670.72, 721.51)	(474.56, 515.09)	(−7.08, −0.18)
Qiaokou	23.94	16.97	−2.73	712.37	504.28	−2.71
	(20.02, 28.60)	(13.76, 21.83)	(−4.85, −0.55)	(689.87, 735.56)	(484.89, 524.99)	(−5.30, −0.04)
Hanyang	24.05	19.35	−2.60	756.31	533.26	−3.49
	(19.41, 29.63)	(15.77, 24.99)	(−4.56, −0.6)	(728.55, 784.98)	(512.84, 555.29)	(−6.03, −0.88)
Wuchang	19.92	14.74	−4.07	592.38	400	−4.70
	(17.02, 23.23)	(12.56, 17.62)	(−6.70, −1.36)	(575.73, 609.44)	(387.88, 412.73)	(−7.68, −1.61)
Qingshan	25.28	18.14	−2.73	710.31	541.71	−2.76
	(20.84, 30.52)	(14.68, 23.72)	(−5.02, −0.39)	(685.51, 735.9)	(520.62, 564.43)	(−5.40, −0.05)
Hongshan	27.07	18.34	−4.76	789.84	503.04	−5.56
	(22.72, 32.27)	(15.52, 21.68)	(−6.77, −2.70)	(765.11, 815.37)	(487.36, 519.22)	(−7.49, −3.59)
Dongxihu	37.28	22.88	−6.11	1182.24	636.13	−6.41
	(29.11, 47.21)	(17.72, 30.39)	(−7.30, −4.91)	(1134.79, 1231.32)	(607.10, 667.13)	(−7.37, −5.45)
Hannan	39.78	17.23	−7.25	1251.97	406.09	−9.68
	(30.79, 50.73)	(12.56, 24.93)	(−10.00, −4.41)	(1199.79, 1305.98)	(382.14, 432.39)	(−12.36, −6.91)
Caidian	39.41	27.14	−1.52	1260.20	732.60	−3.14
	(33.35, 46.32)	(22.56, 32.99)	(−7.79, 5.18)	(1224.53, 1296.71)	(707.26, 759.08)	(−9.44, 3.60)
Jiangxia	37.82	25.54	−4.45	1246.71	759.00	−5.41
	(32.29, 44.06)	(21.33, 30.55)	(−7.08, −1.74)	(1214.14, 1279.96)	(734.82, 783.94)	(−7.97, −2.79)
Huangpi	37.94	21.57	−7.31	1227.15	622.18	−8.76
	(34.00, 42.25)	(18.86, 24.70)	(−9.61, −4.95)	(1204.12, 1250.53)	(606.77, 638.00)	(−11.08, −6.38)
Xinzhou	48.88	32.83	−4.70	1566.99	971.88	−5.93
	(44.06, 54.09)	(29.16, 36.98)	(−6.92, −2.43)	(1539.10, 1595.27)	(950.61, 993.62)	(−8.23, −3.57)

**Table 2:** The effects of population growth, population aging, and the age-specific mortality rate on the change in liver cancer death.

	Change in Liver Cancer Death	Change due to Population Growth	Change due to Population Aging	Change due to Changes in Age-Specific Mortality
Both	−12.42%	9.75%	21.15%	−43.32%
Men	−13.86%	9.13%	19.40%	−42.39%
Women	−8.06%	10.39%	25.54%	−44.00%

## Data Availability

Not applicable.

## Supplementary Materials

The following supporting information can be downloaded at: https://www.mdpi.com/article/10.3390/curroncol30010071/s1, Table S1: ASMR per 100,000 of liver cancer in Wuhan from 2010 to 2019; Table S2: ASYR per 100,000 of liver cancer in Wuhan from 2010 to 2019.
