# Supplementary material for: Secular Trends of Liver Cancer Mortality and Years of Life Lost in Wuhan, China 2010–2019
Source: Curr Oncol. 2023 Jan 9;30(1):938–48. doi: 10.3390/curroncol30010071 (PMC9858443; doi:10.3390/curroncol30010071)
Supplement: Supplementary file 1 [file curroncol-30-00071-s001.zip › curroncol-2066133-supplementary.pdf]

Table S1.ASMR per 100 000 of liver cancer in Wuhan from 2010 to 2019

	Both	Men	Women
2010	30.87	47.15	14.90
2011	29.33	43.62	15.32
2012	28.45	42.29	14.93
2013	28.08	42.94	13.58
2014	27.19	40.68	13.98
2015	24.89	37.73	12.32
2016	22.97	34.73	11.46
2017	23.87	36.47	11.56
2018	20.56	33.05	8.52
2019	20.29	31.13	9.78

Table S2. ASYR per 100 000 of liver cancer in Wuhan from 2010 to 2019

	Both	Men	Women
2010	969.35	1507.56	430.50
2011	907.40	1374.47	441.08
2012	860.72	1310.85	412.84
2013	860.87	1338.43	384.94
2014	812.87	1243.75	382.54
2015	736.38	1145.78	328.40
2016	672.18	1050.56	294.53
2017	694.03	1099.95	290.70
2018	587.37	967.62	212.76
2019	581.82	923.01	243.48
